# Multi-Objective Optimization of Nd: YAG Laser Drilling of Optical-Grade Acrylic Plate Using Taguchi-Based Grey Relational Analysis

**DOI:** 10.3390/ma15248998

**Published:** 2022-12-16

**Authors:** Ming-Jong Tsai, Lung-Fa Wu

**Affiliations:** Graduate Institute of Automation and Control, National Taiwan University of Science and Technology, Taipei 106335, Taiwan

**Keywords:** laser beam machining (LBM), laser drilling, heat-affected zone (HAZ), neodymium-doped yttrium aluminum garnet (Nd: YAG), poly (methyl methacrylate) (PMMA), Taguchi method, grey relational analysis (GRA)

## Abstract

This study proposed an effective method for optimizing laser drilling processing (LDP) by using grey relational analysis (GRA) for multiple performance requirements. First, we developed a system using a Quantel Brilliant Neodymium-doped Yttrium Aluminum Garnet (Nd: YAG) laser with a pulse width of 5–6 ns and F-theta lenses to deliver a focused laser beam with a diameter of 0.2 mm. The developed system was first employed to drill holes in a 3-mm-thick optical-grade acrylic polymethyl methacrylate (PMMA) plate on a safe window with a high optical density and a grade of OD 7+ @ 950~1085 nm. To avoid errors in the experimental data due to unstable power, a laser power (energy) meter was used to measure the energy stability of the Quantel Brilliant Pulse Laser. Given the stability of 5.6%, this is an effective method for LDP. Four control factors were investigated, including laser pulse energy, repetition rate, focusing position offset, and drilling time. Then, nine experiments were performed using the Taguchi method with orthogonal arrays in L_9_ (3^4^). The experimental results with multiple quality characteristics were measured and used to optimize the control factors by using GRA with equal weighting of the four qualities (roundness, Hillock ratio, taper, and HAZ). The results show that A1B3C1D1 is the optimal combination of the control factors, and the maximal variation of 0.406 is obtained from the control factor B (focusing position offset) which has the greatest contribution to the drilling time. We then performed confirmation experiment and obtained a better result from the combination of the control factors, A1B3C1D1. GRA helps us determine the best laser drilling parameters to meet the desired multiple drilling qualities.

## 1. Introduction

Laser beam machining (LBM) is a widely used energy-based non-contact advanced machining process which may be applied to a wide variety of materials. The performance of cutting quality can be improved considerably via an appropriate selection of a laser and operating parameters. Dubey and Yadava reviewed many studies focusing on the development and application of laser beam cutting technology [1].

Nd: YAG laser has less reflection on metal surfaces because of its shorter wavelength, and its high absorptivity enables it to process more reflective materials with relatively less laser energy. However, pulsed Nd: YAG laser produces high laser beam intensity, low mean beam power, narrow heat-affected zone (HAZ), and small recast layer in cutting applications with a high peak power or a shorter interaction time [2,3].

Grey relational analysis (GRA) is an important technique for making predictions and decisions effectively in various manufacturing and product processing areas. Anup Malik et al. [4] used GRA to optimize the input process parameters of the laser-assisted jet electrochemical machining (LA-JECM) and improve machining performance. Taguchi’s method-based design of experiment with L_16_ (4^4^) orthogonal array was employed in the experiments. The optimal parametric combination for multi-response optimization was identified based on the collective implementation of the Taguchi methodology and GRA during the micro-drilling of Inconel-718. Thus, LA-JECM was developed and used in subsequent experimental investigation [4].

In laser material processing (LMP), the heat-affected zone (HAZ) of the material occurs when it is subjected to long-duration or high-energy laser exposure. This, in turn, affects the quality of drilling when the laser processing time is insufficient. However, a longer processing time results in a large HAZ and other effects [5]. When the laser energy is too high (e.g., when it starts burning black spots on the material), an acrylic (PMMA) [6] plate target can expand rapidly and undergo undesirable modifications, such as crazing. The parameters associated with laser processing, their effects on the HAZ, surface roughness and the depth, width, and shape of the drilled hole have been investigated [5].

Since laser beam machining (LBM) had no effects on tool wear and cutting force or vibrations during cutting, laser beam drilling has been proposed as an alternative to improving traditional drilling [7]. In one study, variance analysis was used to investigate the impact of scanning speed and frequency on macro-geometric parameters, surface quality, and defects (tapers and heat-affected zones). Moreover, to identify drilling wall problems, stereoscopic optical microscopes (SOMs) and scanning electron microscopes (SEMs) were used. This experimental method revealed the conditions to minimize deviations, defects, and damage in hole drilling [7].

The theory of roundness is an important concept in many engineering fields. The relevant roundness parameters are defined in the ISO 12181-1 [8] and used as standard filters in the ISO 12181-2 [9]. When determining a roundness profile for the cross-sections of large flexible rotors, such as paper machine rolls, several conditions can restrict the available roundness measurement methods. Rotors may be too large to be placed onto precision spindles, and, under rotation, the cross-sections’ center point movement can be unpredictable and unrepeatable [9]. When using a multi-probe roundness measurement frame position, the resulting error may cause the angle of the entire probe to deviate from the target angle. In fact, the angle of the probe can be accurately determined by its cross-correlation after being placed in the measurement frame [10]. Determining the center point coordinates and the roundness error is essentially a problem with three degrees of freedom. Thus, they have to be measured by at least three probes to become independent. Measurement with one probe is sufficient only when the measurements are performed on a precision spindle with a roundness measuring instrument or in a coordinate measuring machine [11].

K. Pratap Singh et al. [12] conducted L_9_ orthogonal array-based experiments. Since then, hole circularity and taper angle have been measured for L_9_ with different combinations of input laser drilling parameters. Furthermore, the Taguchi optimization technique has been implemented for hole circularity and taper angle, and the non-uniform dimensioning hole has been employed due to the converging-diverging nature of the laser beam. Hence, a lower value of the taper angle gives better hole circularity [12].

Pan LK et al. [13] demonstrated that a hybrid approach combining the Taguchi method and principal component analysis can be applied to laser beam cutting to optimize input parameters and their influence on multiple quality characteristics. Among them, the GRA proposed by Deng [14] in 1989 is one of the foremost techniques applied when the nature of information is incomplete and uncertain. As implied by its name, GRA can effectively optimize complex interrelationships. It has been widely used in evaluating available information and complex projects [13].

Li et al. [15] employed Taguchi’s method and applied a complicated score transformation to transfer six cutting-quality characteristics of each experiment into a single cutting-quality score. However, the transferred equations could not easily indicate the importance of each required quality in the experiments. Therefore, Tsai et al. [16] employed a Taguchi’s method-based GRA approach to directly integrate six laser-cutting quality characteristics of each experiment into a grey relational grade (GRG). The actual cutting results could be immediately known by the obtained grades [16].

Kumar Singh Yadav et al. [17] presented an experimental study and a machining parameter design of electro-discharge diamond grinding (EDDG). The EDDG setup was designed and fabricated, and the experiments were conducted on high-speed steel (HSS) workpieces under varying currents, pulse-on time, duty factor, and wheel speed. The settings of the machining parameters were determined using the Taguchi experimental design method [17].

Das et al. [18] proposed a Taguchi-Based grey relational analytical method to determine the optimal processing parameters of Ti6Al4V. The machining forces, including longitudinal force (F_x)_, radial force (F_y_), tangential force (F_z_), surface roughness, and material removal rate (MRR), were measured during the facing operation. The effects of three process parameters, including cutting speed, tool feed, and cutting depth, were studied on the matching responses. The orthogonal design of the experiment (Taguchi L_9_) was adopted to execute the process parameters at each level. The GRA optimization approach was applied to validate the process output parameters. It was found that the hardness of Ti6Al4V MMC was 59.4 HRA and the composition of the material remained the same after the milling operation [18].

C. Sarala Rubi et al. [19] investigated the effects of machining parameters during the drilling of LM 6/B4C composite materials. The optimization process aimed to attain the lowest thrust force (TF), surface roughness (SR), and burr height (BH). The feed rate (F), the spindle speed (S), the drill material (D), and the reinforcing percentage (R) were determined. Finally, the predicted GRG was 0.846, whereas the experimental GRG was 0.865, with a 2.2% error indicating that the optimization process was valid [19].

Due to the localized and non-contact process, the use of lasers for cutting material can result in precise cuts with a small heat-affected zone (HAZ). One study used GRA to determine a single optimized set of cutting parameters for three different thermoplastics. The optimized processing parameters were found at low laser power (200 W), high cutting speed (0.4 m/min), and low compressed air pressure (2.5 bar) [20].

Prasanth Achuthamenon Sylajakumari et al. [21] studied the optimization of wear parameters using the Taguchi method with grey relational analysis and their effect on dry sliding wear performance of AA6063/SiC co-continuous composite. A Taguchi L_9_ orthogonal array was designed, and nine experimental runs were performed based on the planned experiments. The confirmation experiments conducted using the optimal parameters indicated an improvement in quality [21].

A new optimization strategy was used to investigate the effects of processing parameters and cryogenic treatment on cutting force and surface roughness in the milling of Ti6AI4V alloy, and it was proposed to simultaneously optimize the response parameters by EKİCİ, E. et al. The effects of cutting speed, feed rate, and the treatments applied to the tools were evaluated using the Taguchi method and grey relational analysis. The control factors in the experiments performed under dry cutting conditions were based on two different cutting speeds and three different feed rates and tool properties. It was observed that the cutting force values decreased with increased cutting speed and significantly increased parallel to the feed rate. In terms of surface roughness, they were observed to change based on the cutting parameters. The optimal machining conditions were determined as A1B3C2 after the grey relational analysis was performed on both responses [22].

GRA-based Taguchi methods for the optimization of submerged arc welding (SAW) process parameters in hardfacing were adopted to solve the SAW process with multiple weld qualities in one study. A grey relational analysis was used for the multiple characteristic optimization. Then, the optimal process parameters were determined [23].

The research by Md. Nahid Pervez et al. [24] investigated the influence and optimization of the factors for a non-formaldehyde resin finishing process on cotton fabric using a Taguchi-based grey relational analysis. An L_27_ orthogonal array was selected for five parameters with three levels by applying Taguchi’s design of experiments. The Taguchi technique was coupled with a grey relational analysis to obtain a GRG for evaluating multiple responses. The results showed an improved thermal stability and confirmed the presence of well-deposited resin on the optimized fabric surface [24].

Wanqin Zhao et al. [25] compared the results of micro-deep hole drilling by picosecond ultra-short pulse laser in four metals, including copper, aluminum alloy, titanium alloy, and nickel alloy. Destructive testing was performed to measure the depth, shape, and side-wall morphology of the micro-deep holes. The diameters and depths of the micro-deep holes in the four metals ablated using the same processing parameters were compared. The influence mechanisms, especially the side-wall morphology, such as the thresholds, conical emission, self-cleaning effect, physical properties of metals, energy transmission, plasma effect, and thermochemical reaction, were discussed. [25].

In the studies by Pu et al. [26], the effects and optimization of laser power, spindle speed, feed rate, and cutting depth on surface roughness and work hardening of LAM Si3N4 were systematically studied by using grey relational analysis coupled with the Taguchi method. The results showed that the combination of machining parameters determined the material removal mode at the material removal location, and this then affected the surface quality [26].

An analysis of the GRG in one study indicates that the parameter significance and the optimal parameter combination for a laser-cutting process can be identified. The analytical results from two confirmation experiments using the optimal parameters confirmed that the laser-cutting technology could be effectively applied to cut substrates into special shapes [27].

Laser drilling has swiftly become an economical and well-regulated substitute for conventional hole drilling methods. The circularity of drilled holes at the entry and exit and the taper are very important attributes that influence the quality of a drilled hole by a laser beam. For this reason, an experimentation based on central composite design was performed on austenitic stainless steel to examine the effect of laser parameters, such as lamp current, pulse frequency, gas pressure, and pulse width, on the quality of drilled holes [28].

The experimental parameters and their levels were tested in the study by Gonfa et al. [29] at three different stirring speeds (350, 450, and 550 rpm), three different stirring times (5, 10, and 15 min), three different weight percentages of SiC (0, 5, 10 wt.%) and three different weight percentages of MoS2 (0, 2, 4 wt.%). Nine samples were fabricated by stir casting using the Taguchi method L_9_ orthogonal arrays. Composite hardness, tensile strength, and wear rate were investigated using Taguchi’s signal-to-noise ratio as a single response characteristic, and hybrid Taguchi–grey relational analysis (HTGRA) was used to study and analyze them as a multi-response characteristic. The results revealed that the addition of SiC in the composite produced better hardness, tensile strength, and wear rate [29]. Tsai et al. presented Optimization of laser-cutting parameters for QFN packages by utilizing artificial neural networks and genetic algorithm [30]. A more detailed cutting quality can be predicted by using the proposed method. 

Laser material processing (LMP) is used to replace traditional material processing techniques, such as material cutting, thin plate drilling, and surface processing. The early laser plasma is used as a cleaning material and the Raman spectroscopy is used for material identification. Laser plasma is produced when a high-energy laser beam meets matter, leading to a rapid increase in the temperature of the material. The energy of a pulsed laser can be varied to alter the cavity gap created by the laser plasma. The working distance is set to a value to obtain a negative drill-hole divergence angle. The characteristics of the laser plasma can be varied by changing the laser energy, thereby broadening its potential applications. A 3-mm-thick, optical-grade acrylic (PMMA) plate was drilled using a neodymium-doped yttrium aluminum garnet (Nd: YAG) pulse laser in one study, and the plasma effect between the focusing position offset and taper were investigated [5]. In this study, the Nd: YAG pulse laser, including a self-designed optical alignment system, was used for drilling an acrylic (PMMA) plate of high optical density grade. The highlight of the current work is described below. The laser plasma generated by the Nd: YAG pulse laser can produce a cavity gap located on the focusing position of the acrylic (PMMA) plate so that the taper angle and the divergence angle can be easily distinguished. Nine sets of experiments were performed by using the Taguchi method with orthogonal arrays in L_9_(3^4^). The experimental results with multiple quality characteristics are measured and used to optimize the control factors by using GRA with equal weighting factors of four qualities to find the best quality processing parameters.

## 2. Materials and Laboratory Tools

In this study, we used an acrylic or poly (methyl methacrylate) PMMA plate (Model P5P05) with Laser Safe Flat Window and Visible Light Transmission (VLT) by LASERVISION [6], which is an optical grade safety window 7+ with high optical density (OD) and a thickness of 3 mm. The PMMA plate is lightweight, easy to fabricate, and has high chemical stability and mechanical strength [6].

**Figure 1:**
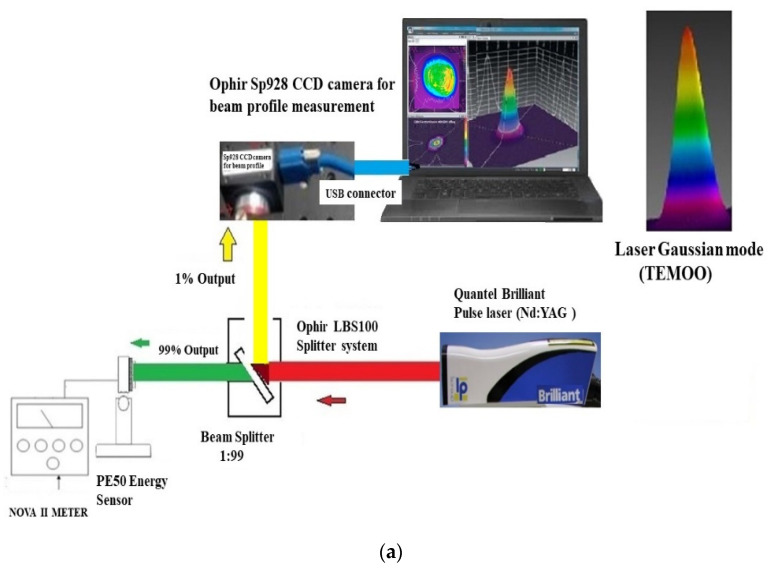

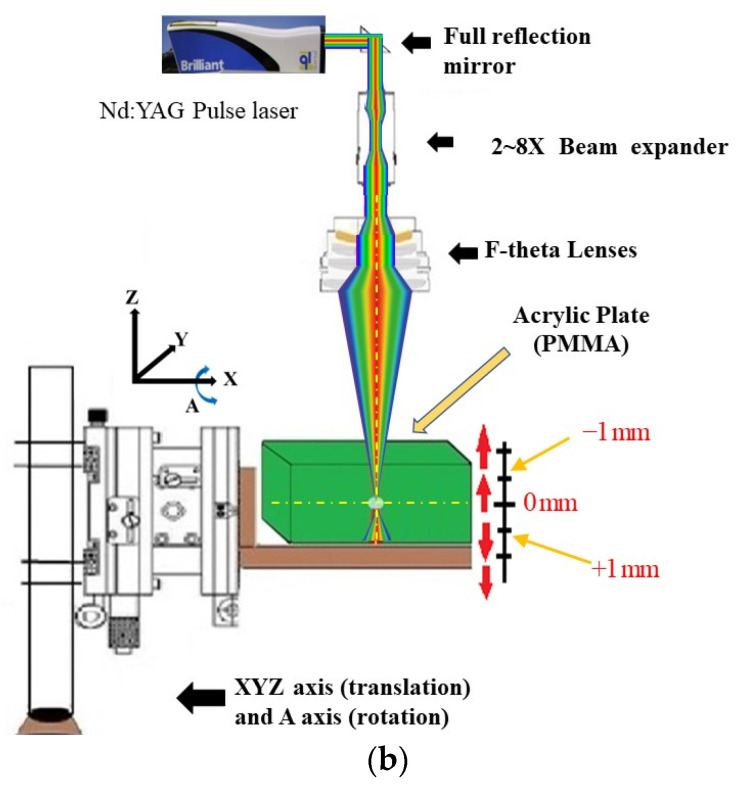
Experimental setup: (**a**) laser energy and beam profile measurement, and (**b**) schematic of laser beam alignment and focusing position adjustment.

A Nd: YAG pulse laser (Quantel Brilliant [31]), which has a maximum energy of 360 mJ per pulse, was designed to drill the PMMA plate. The used beam splitter (Ophir LBS-100) has a ratio of 1%:99% [32]. About 99% of the laser energy is split for the laser energy measurement by an energy sensor (Ophir PE50BB-DIF [32]) and a display meter (Ophir NOVAII) [32]. The other 1% of the laser energy is used for the beam profile measurement using a CCD (Ophir SP928 [32]), which is connected to a computer through a USB interface, as shown in Figure 1a. The laser beam profile with an approximate Gaussian mode (TEM 00 mode) was confirmed using the Ophir BeamGage software [33].

To avoid measurement uncertainty due to unstable power, a laser power meter was used to measure power stability in which the laser power was set at 1.053 W and measured for one hour. As shown in Table 1, the result shows a stability of 5.6%, making it an effective method. The comprehensive specifications of the used Nd: YAG laser are shown in Table 1.

The laser’s light source is a high-energy pulse laser (145.0 mJ/pulse, generating 3000 pulses in 7 min). The energy density per pulse produced is 522.98 J/cm^2^. In the system setup, a beam expander (LINOS Model: 4401-256-000-20 [34]) and an F-theta lens (LINOS Model: 4401-508-000-26 [35]) are combined to adjust the laser beam shape with a spot size of a 0.2 mm diameter, as shown in Figure 1b. It is necessary to make sure that the incident beam is focused on the center of the F-theta lenses. A motorized XYZ and rotation stage is used to carry the drilled PMMA plate. Finally, the focusing position of the laser beam is located at the center of the PMMA plate with thickness of 3 mm. During the experiment, the Z-axis can be adjusted forwardly and backwardly to obtain and focus offset. The schematic of the laser beam alignment and focusing position adjustment is shown in Figure 1b.

The Taguchi method with orthogonal arrays and GRA were employed to find the controlled parameters for the best drilling quality. The main four control factors discussed in this study are laser pulse energy (A), focusing position offset (B), drilling time (C), and repetition rate (D). Each factor has three levels (Level 1, Level 2, and Level 3), which are shown in Table 2. Factor A (laser energy) has three levels: 75, 110, and 145 (mJ/pulse). Factor B (focusing position offset) may be (−1 mm), (0 mm), or (+1 mm) from the center of the PMMA plate. Factor C (drilling time) may be set as 3 min, 5 min, and 7 min. Factor D (repetition rate) may be set as 1.8 kHz, 3.0 kHz, and 4.2 kHz. The definition of the terms and measurement methods will be described in the next section. These control factors of the orthogonal arrays were used to optimize performance characteristics related to laser drilling on the PMMA plate, which included roundness, hillock width, taper, and HAZ qualities.

It has been shown that limiting laser energy to between 75 and 145 mJ/pulse can avoid the phenomenon of excessive HAZ caused by high energy and low drilling quality caused by low energy. The focusing position offset between +1 and −1 mm is suitable for observing taper and can narrow the range in factor design [5].

When applying the Taguchi-based experiment design of L_9_ (3^4^), there are nine experiments (runs) to be conducted with the parameters shown in Table 3. After the experiments have been conducted, the obtained experimental results with multiple quality characteristics are used to optimize the control factors by using GRA with equal weighting of the four qualities.

## 3. Qualities and Measurement Methodology

The main qualities after laser drilling on the PMMA plate were confirmed by the specified measurement data. The shape of the drilled hole might be irregular. To facilitate the definition of quality, the characteristics of the drill holes were classified into the following categories. The measurement methods and quality calculation will be described in this section.

### 3.1. The Roundness of the Drilled Hole

**Figure 2:**
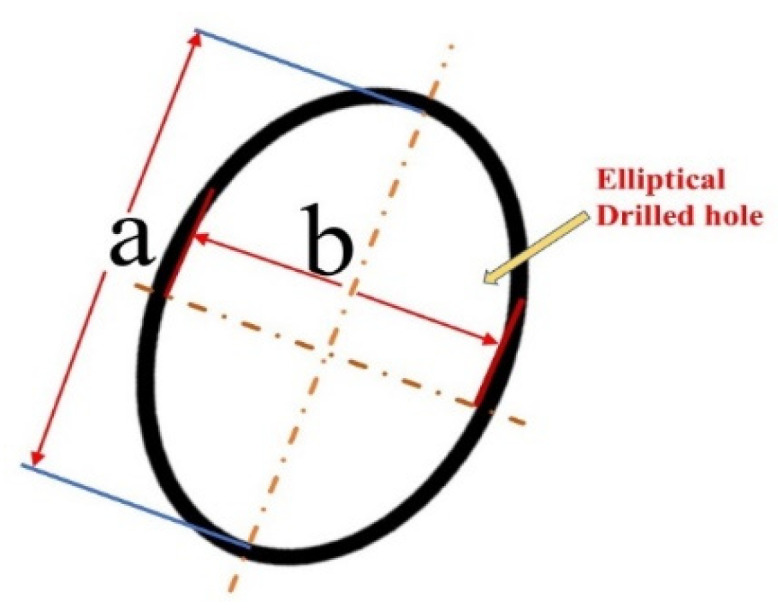
Elliptical approximation of a drilled hole at the entrance and the exit side.

To evaluate the roundness of a laser-drilled hole, the irregular hole is considered an elliptical shape, as shown in Figure 2. Both elliptical shapes at the entrance side (*E_en_*) and the exit side (*E_ex_*) are considered. The roundness at the entrance side is calculated from Equation (1).

(1) $$E_{en}^{}=\frac{b_{e}^{}}{a_{e}^{}}$$

where *b_e_* is the shorter or minor elliptical diameter at the entrance side (mm) and *a_e_* is the longer or major elliptical diameter at the entrance side (mm).

Similarly, the roundness of a drilled hole at the exit side is calculated from Equation (2).

(2) $$E_{ex}^{}=\frac{b_{x}^{}}{a_{x}^{}}$$

where *b_x_* is the shorter or minor elliptical diameter at the exit side (mm) and *a_x_* is the longer or major elliptical diameter at the exit side (mm).

After the roundness of a drilled hole has been calculated from Equations (1) and (2), the average roundness *E_avg_* can be obtained from Equation (3).

(3) $$E_{avg}^{}=\frac{\left(E_{ex}^{}+E_{en}^{}\right)}{2}$$

where *E_ex_* is the elliptical roundness at the exit side and *E_en_* is the elliptical roundness at the entrance side.

An average roundness of 1, which is the possible maximum, indicates the best quality. For an ideal circle, the larger the roundness, the better the drilling quality.

Since the Nd: YAG pulse laser has a small amount of energy, it has little effect on the temperature [6,7,8,9,10,11]. In the solid color picture (top view) of the drilled PMMA plate sample, we can see the depth of the drilling cut and a cutting plane intersecting the flat body, which looks like a two-dimensional picture with a three-dimensional spatial shape. From the brown color appearance, we can locate the HAZ range and calculate the true roundness by the length and distance known from the geometry.

### 3.2. The Hillock Ratio

**Figure 3:**
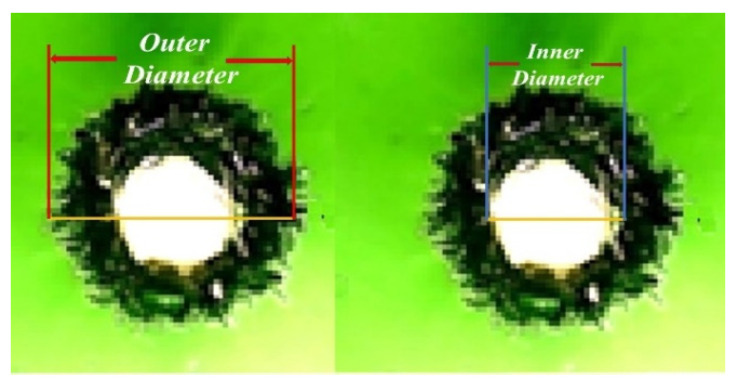
Hillock measurement of the inner and outer diameters.

To evaluate the Hillock ratio of laser-drilled holes, irregular holes are considered to be shapes with Hillock width. The Hillock width is calculated from the inner and outer diameters at the entrance side (*Hill_en_*) and the exit side (*Hill_ex_*), which is shown in Figure 3. When a drilled hole is measured at the entrance side, the outer diameter is *O_e_* and the inner diameter is *I_e_*. Both the *O_e_* and *I_e_* measurements were performed at two cross-sections at an angle of 0–180°and 90–270°, and the average value was calculated. Using the obtained measurement, the Hillock ratio *Hill_en_* at the entrance side is calculated from Equation (4).

(4) $$Hill_{en}=\left(\frac{O_{e}^{}-I_{e}^{}}{\;O_{e}^{}}\right)$$

where *O_e_* is the outer diameter at the entrance side and *I_e_* is the inner diameter at the entrance side.

Similarly, we measured the drilled hole at the exit side using the outer diameter, *O_x_*, and the inner diameter, *I_x_*. Both the *O_x_* and *I_x_* measurements were performed at two cross-sections with an angle of 0–180°and 90–270°, and the average value was calculated. The Hillock ratio at the exit side (*Hill_ex_*) is obtained by applying Equation (5).

(5) $$Hill_{ex}=\left(\frac{O_{x}^{}-I_{x}^{}}{\;O_{x}^{}}\right)$$

where *O_x_* is the outer diameter at the exit side and *I_x_* is the inner diameter at the exit side.

The average Hillock ratio (*Hill_avg_*) is calculated by applying Equation (6).

(6) $$Hill_{avg}=\left(\frac{Hill_{en}^{}+Hill_{ex}^{}}{2}\right)$$

where *Hill_en_* is the Hillock ratio at the entrance side and *Hill_ex_* is the Hillock ratio at the exit side. An average Hillock ratio of 0, which is the possible minimum, indicates the best quality. The smaller the hillock ratio, the better the drilling quality.

### 3.3. The Taper Quality

**Figure 4:**
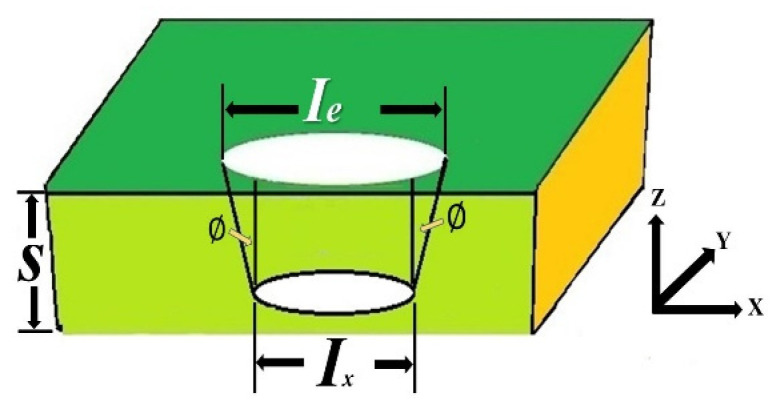
Schematic of average taper (*T_tap_*) of a drilled hole.

The taper quality is evaluated as shown in the schematic of taper measurement for a drilled hole in Figure 4, with taper being defined in Equation (7).

(7) $$T_{tap}=\left(\frac{I_{e}-I_{x}}{2\cdot S}\right)$$

where *I_e_* is the inner diameter at the entrance side and *I_x_* is the inner diameter at the exit side. *S* is the thickness (3 mm) in this study [12].

### 3.4. The Heat-Affected Zone (HAZ)

**Figure 5:**
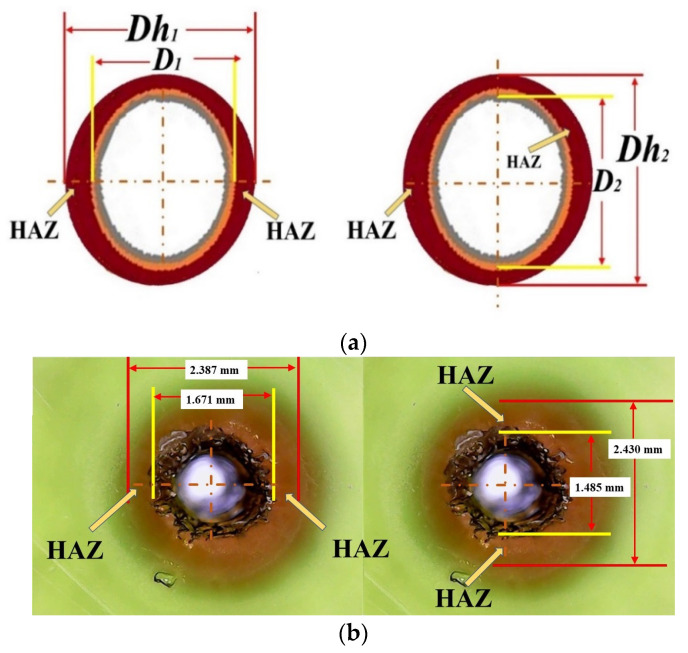
(**a**) Schematic of HAZ measurement. (**b**) Actual photo of HAZ measurement.

The HAZ is evaluated as shown in the schematic in Figure 5a. The actual measurement photo is shown in Figure 5b. The average of HAZ at the entrance side and the exit side is shown in Appendix A Table A4. Equation (8) is used to calculate the average *HAZ_avg_*, which is shown in the right column of Appendix A Table A4. The HAZ measurements were performed at two cross-sections with an angle of 0–180°and 90–270°, and the average value was calculated from the entrance and exit sides. The overall HAZ is obtained by applying Equation (8).

(8) $$HAZ_{avg}^{}=\frac{\left(Haz_{en}^{}+Haz_{ex}^{}\right)}{2}$$

where *Haz_en_* is the average HAZ (mm) at the entrance side and *Haz_ex_* is the yellow average HAZ (mm) at the exit side.

### 3.5. Grey Relational Generation

Notably, GRA utilizes the mathematical method for analyzing correlations between the series comprising a grey relational system, and it thereby determines the difference in contribution between a reference series and each compared series. The compared series are alternative vectors created from the sets based on attribute characteristics, which are the larger-the-better (LTB), the smaller-the-better (STB), or optimization of specific values between the maximal and minimal values of an attribute. By applying a GRA algorithm, the GRGs of different series can be used to rank various alternatives, where higher values indicate superior alternatives [27].

Data pre-processing is the first step in the procedure for using GRA. Data pre-processing involves transforming an original sequence into a comparable sequence. Experimental results are, thus, normalized in a range from 0 to 1. Equation (9) shows the calculations for the LTB case; Equation (10) shows those for the STB case; and Equation (11) shows those for the case in which a definite target value must be achieved [27].

(9) $$X_{i}^{*}\left(k\right)=\frac{X_{i}^{\left(0\right)}\left(k\right)-{min}_{all\;(i)}X_{i}^{\left(0\right)}\left(k\right)}{{max}_{all\;(i)}X_{i}^{\left(0\right)}\left(k\right)X_{i}^{\left(0\right)}\left(k\right)-{min}_{all\;(i)}X_{i}^{\left(0\right)}\left(k\right)}$$

(10) $$X_{i}^{*}\left(k\right)=\frac{{max}_{all\;(i)}X_{i}^{\left(0\right)}\left(k\right)-X_{i}^{\left(0\right)}\left(k\right)}{{max}_{all\;(i)}X_{i}^{\left(0\right)}\left(k\right)-{min}_{all\;(i)}X_{i}^{\left(0\right)}\left(k\right)}$$

(11) $$X_{i}^{*}\left(k\right)=1-\frac{\left|X_{i}^{\left(0\right)}\left(k\right)-OB\right|}{{max}_{all\;(i)}\left\{O1,O2\right\}}$$

where $\;X_{i}^{*}$(*k*) is the *i*^th^ grey datum following grey generation for experiment *k*; $X_{i}^{\left(0\right)}$(*k*) is the original *i*^th^ quality datum of experiment k; *max_all_*_(*i*)_$X_{i}^{\left(0\right)}$(*k*) is the maximal value in the original sequence; *min_all_*_(*i*)_$X_{i}^{\left(0\right)}$(*k*) is the minimal value in the original sequence; and OB is the target value, with *O*_1_ = *max_all_*_(*i*)_$X_{i}^{\left(0\right)}$(*k*) − *OB* and O_2_ = *OB* − *min_all_*_(*i*)_$X_{i}^{\left(0\right)}$(*k*).

The roundness is obtained by applying Equation (9) in the LTB case. The Hillock and HAZ result in a minimal roundness of 0, which indicates the best quality. The GRG calculation of HAZ and Hillock are obtained by applying Equation (10) in the STB case. In the calculation of taper, it is expected that the entrance diameter is equal to the exit diameter. The GRG calculation of taper quality is obtained by applying Equation (11) in the nominal-the-best (NTB) case, where the zero value is the best among the maximal positive and the minimal negative.

### 3.6. Grey Relational Coefficient and Grey Relational Grade (GRG)

Following data pre-processing, a grey relational coefficient is calculated to express the relationship between the ideal and the actual normalized experimental results. The grey relational coefficient for the four qualities in the nine experiments is calculated by applying Equation (12).

(12) $$\gamma_{i}^{}\left(k\right)=\frac{\Delta_{min}\;+\zeta \Delta_{max}}{\Delta_{i}\left(k\right)+\zeta \Delta_{max}}\;k\;=\;1,2\;,...,9;\;i\;=\;1,2\;,3,4$$

(13) $$\Delta i\left(k\right)=\left|\left|X_{i}^{*}\left(0\right)-X_{i}^{*}\left(k\right)\;\right|\right|$$

(14) $$\Delta_{max}\;=\begin{array}{c} max\\ \forall \;j\in i \end{array}\begin{array}{c} max\\ \forall \;k \end{array}\left|\left|X_{i}^{*}\left(k\right)-X_{j}^{*}\left(k\right)\right|\right|$$

(15) $$\Delta_{min}\;=\begin{array}{c} max\\ \forall \;j\in i \end{array}\begin{array}{c} max\\ \forall \;k \end{array}\left|\left|X_{i}^{*}\left(k\right)-X_{j}^{*}\left(k\right)\right|\right|$$

where Δ_0_*_i_(k)* is the deviation sequence between the reference sequence $X_{i}^{*}$(0) and $X_{i}^{*}$(*k*), and ζ is the distinguishing or identification coefficient (ζ ε [0,1]) [14], which is set to be 0.5 in general case.

However, the importance of each relational coefficient to the final quality is not the same in terms of system requirements. When the weighting factors are not equal, the overall GRG can be obtained by applying Equation (16).

(16) $$\gamma \left(k\right)=\sum_{i=1}^{4}{\;\gamma }_{i}^{}\left(k\right)*w_{i}^{}\;k\;=\;1,2\;,...,9,$$

where γ(*k*) is the GRG of each experiment obtained by taking the average of the weighting grey relational coefficients; *w_i_* is the weighting factors for each quality; and *i* = 1, 2, 3, 4 is the quality number. The overall GRG quality may be affected by the weights of each equality. The GRG shows the important relationships among the sequences and indicates their degree of impact. The average response of each level effect for all factors in all experiments, as shown in Table 2, is discussed, and the best combination of levels for each factor can be obtained as a result. A detailed description will be given in Section 4.

## 4. Results and Discussion

In this study, the four control factors are laser energy, focusing position offset, drilling time, and repetition rate. The four qualities are roundness, Hillock ratio, taper, and HAZ. When applying the Taguchi-based experiment design to L_9_ (3^4^), nine experiments (runs) were conducted with the parameters shown in Table 3. Then, the four qualities are obtained, as shown in Appendix A Table A1, Table A2, Table A3 and Table A4. Next, we used the experimental results with multiple quality characteristics to optimize the control factors by using the GRA with equal weighting.

### 4.1. Quality Data Measurement and Calculation

First, to evaluate the roundness, four data *ae, be, ax,* and *bx* are measured according to Figure 2. The measured data for roundness with an elliptical approximation are shown in Appendix A Table A1. Equations (1) and (2) are used to calculate the roundness from the major and minor diameters of the elliptical shape at the entrance side and the exit side, respectively. Finally, Equation (3) is used to calculate the average roundness. The experimental results show that the maximal roundness is 0.921 and the minimum is 0.684.

To evaluate the second quality, Hillock ratio, the inner and outer diameters at the entrance side (*O_e_*) and at the exit side (*O_x_*) are measured according to Figure 3 and shown in Appendix A Table A2. Equations (4) and (5) are used to calculate the Hillock ratio (Hill_en_ and Hill_ex_) at the entrance side and the exit side, respectively. Finally, Equation (6) is used to calculate the average Hillock ratio, as shown in the right column of Appendix A Table A2. The experimental results show that the maximal average Hillock ratio is 0.680 at Run 7 and the minimum is 0.253 at Run 9. This is because Run 7 has the maximal applied laser energy.

To evaluate the third quality, taper, the average values of the drilled hole for the inner diameter at the entrance side (*I_e_*) and the inner diameter at the exit side (*I_x_*) are measured and averaged based on Figure 4. The average data of the taper at the entrance and exit sides are shown in Appendix A Table A3. Finally, Equation (7) is used to calculate the average taper (*T_tap_*), as shown in the right column of Appendix A Table A3. The experimental results show that the maximum of taper is 0.1348 at Run 8 and the minimum of taper is −0.0049 at Run 3.

To evaluate the fourth quality, *Haz_en_* and *Haz_ex_* are measured at the entrance side and the exit side, respectively, and averaged based on Figure 5a. The average data of HAZ at the entrance and exit sides are shown in Appendix A Table A4. Equation (8) is used to calculate the average HAZ, which is shown in the right column of Appendix A Table A4. The experimental results show that the maximum of HAZ is 7.0 mm at Run 7 due to the maximal applied laser energy.

The results of the four qualities above are shown in Table A1, Table A2, Table A3 and Table A4 and integrated into Table 4, in which the maximal data for each quality are provided in the first row as a reference. Using Equation (9) to Equation (11), the grey relational generation of multiple qualifiers can be calculated as shown in Table 5. Firstly, Equation (9) is used to calculate roundness with LTB, as shown in the left column in Table 5. The calculated results show that the maximum roundness is 1.000 at Run 6 which has the best roundness among all runs. Secondly, Equation (10) is used to calculate the Hillock ratio with STB, as shown in the second column in Table 5. The calculated results show that the maximum is 1.000 at Run 9 which has the best Hillock ratio among all runs. Thirdly, Equation (11) is used to calculate taper with NTB, as shown in the third column in Table 5. The calculated results show that the maximum of taper is 1.000 at Run 3 which has the best taper among all runs. Fourthly, Equation (10) is used to calculate HAZ with STB, as shown in the right column in Table 5. The calculated results show that the maximum of HAZ is 1.00 mm at Runs 1, 3, and 6, which have the best values for HAZ among all runs.

The deviation sequence from the grey relational generation of multiple qualities is calculated in Table 5, and the maximal grey values for each quality are provided in the first row as a reference. To obtain the GRG, the grey relational coefficients for the four qualities in the nine experiments are calculated by applying Equation (12). The deviation sequences of multiple qualities can be obtained as shown in Table 6 by applying Equation (13). The maximal and minimal values at the bottom of Table 6 are calculated by applying Equations (14) and (15). According to Equations (12)–(15) and Table 6, the grey relational coefficients for the four qualities in the nine experiments are obtained as shown in the left four columns of Table 7.

### 4.2. Response Table for the GRG

The first quality is the calculated result of roundness, which shows the maximum at Run 2 and the minimum at Run 6. The second quality is the calculated result of Hillock ratio, which shows the maximum at Run 7 and the minimum at Run 9. The third quality is the calculated result of taper, which shows the maximum at Run 8 and the minimum at Run 3. The fourth quality is the calculated result of HAZ which shows the maximum at Run 8 and the minimum at Runs 1, 3, and 6.

Finally, Equation (16) is used to calculate the ranking for the overall GRG of all qualities, which may have equal weighting factors. The overall GRG and the rank are shown in the right two columns of Table 7. The calculated results show that the maximum grade of rank 1 is 0.968 at Run 6 and the minimum grade of rank 9 is 0.404 at Run 7.

**Figure 6:**
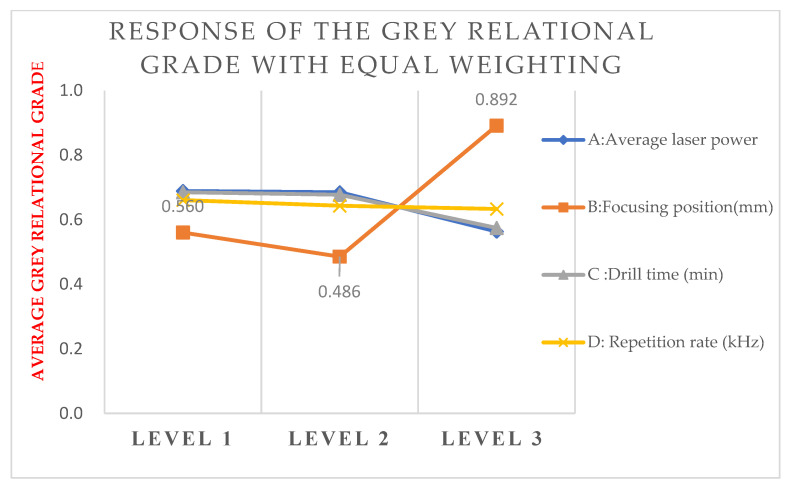
Response of the GRG with equal weighting factors.

To investigate the level effect of each factor, the average response is calculated from the GRG with levels 1, 2, and 3 for all control factors A, B, C, and D. The calculated responses are shown in Table 8 and Figure 6. For the first control factor A, the calculated results show the maximal level effect is 0.689 for laser energy, which is at level 1. For the second control factor B, the calculated results show the maximal level effect is 0.891 for the focusing position offset, which is at level 3. For the third control factor C, the calculated results show the maximal level effect is 0.685 for drilling time, which is at level 1. For the fourth control factor D, the calculated results show the maximal level effect is 0.660 for the repetition rate, is which is at level 1.

Therefore, the best combination of the control factors is A1B3C1D1, as shown in Table 8 and Figure 6. To investigate the contribution of the control factors, the level effect between the maximal and minimal GRGs is calculated as shown in the right column of Table 8. The data show that the maximal variation of 0.406 is from control factor B, which has the greatest contribution to the drilling quality based on equal weighting factors. In Table 7, Runs 3, 6, and 9 with level 3 for the control factor B have the top three high grades among the nine experiments. Therefore, the focusing position offset of −1 mm provides the best results compared to the other two levels. 

### 4.3. Confirmation Experimental Results

A confirmatory experiment was performed with the best combination of the control factors, A1B3C1D1. Then, comparisons were conducted to verify the effectiveness of the proposed method. Factor A laser pulse energy is 75 mJ per pulse; factor B the focusing position offset is −1 mm; factor C the drilling time is 3 min; and factor D the repetition rate is 1.8 kHz. After the confirmation experiment was performed by using the above parameters, the measured data and multiple quality calculation are obtained from Equations (1)–(8), as shown in Appendix A Table A5, Table A6, Table A7 and Table A8.

**Figure 7:**
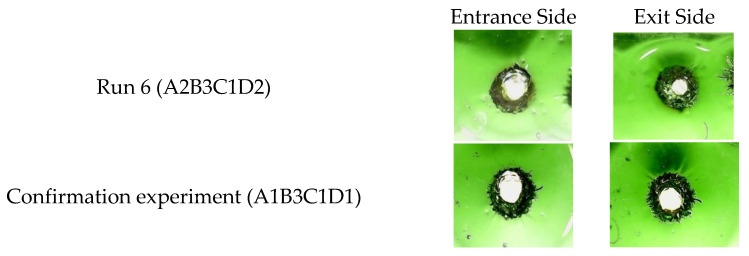
Comparison of the drill photos from the results with optimal combination (A1B3C1D1) and the combination used in Run 6 (A2B3C1D2).

The comparison of the confirmation experiment (A1B3C1D1) and the original best result (A2B3C1D2 at Run 6) are shown in Table 9. The confirmation results show that the roundness value is 0.938, the Hillock ratio value is 0.246, the taper value is −0.0036, and the average HAZ value is 0.00. It can be clearly seen that the results of the confirmation experiment (A1B3C1D1) are better than those of Run 6 (A2B3C1D2). Figure 7 shows real comparative photos of the laser drilled holes to demonstrate the quality improvement and the effectiveness of the used Taguchi-GRA method.

## 5. Conclusions

A Nd: YAG pulse laser system for drilling holes on 3-mm-thick optical-grade PMMA plates is studied in this paper. The laser energy, focusing position offset, drilling time, and repetition rate are employed to explore their effects on multiple qualities upon performance, which include the HAZ, drill roundness, Hillock ratio, and taper. A Taguchi-based GRA method is used to obtain the optimal control factors for multiple qualities. The conclusions of this study are described below:

1. The developed Nd: YAG pulse system is the first to be employed to drill holes in a 3-mm-thick optical-grade acrylic polymethyl methacrylate (PMMA) plate on a safe window with high optical density and a grade of OD 7+ @ 950~1085 nm. This study presents the unique design of the Nd: YAG pulse laser in which the beam profiler and power stability are measured and confirmed to obtain a laser beam with approximate Gaussian mode.
2. From the experimental results, the focusing position offset has the greatest contributions to the multiple qualities. A focusing position offset of −1 mm (Level 3) provides the best results compared to the other two levels.
3. Based on the qualities calculated with equal weighting factors of 25%, the obtained best combination is A1B3C1D1 in which factor A (laser energy) is at level 1, factor B (focusing position offset) is at level 3, factor C (drilling time) is at level 1, and factor D (repetition rate) is at level 1. To verify the validity of the proposed Taguchi-GRA method, confirmation experiments are performed and better results are obtained.
4. By using the Taguchi method and GRA, the optimal values of the factors can be determined in a short time. Further applications can be carried out for different quality requirements if necessary.
5. The Nd: YAG pulse laser in this experiment is suitable for use on objects with smaller size and thinner thickness. In the future, to process objects with larger thickness, it is only necessary to change the Nd: YAG pulse laser source to higher energy and add a nozzle with a jet flow control. This combination could be flexible and feasible for LMP applications.
6. In addition, a Harmonic Generator can be installed at the laser outlet to change the wavelength. For example, a SHG (Second Harmonic Generator −2ω) with a laser of 1064 nm can obtain a laser wavelength of 532 nm, which can obtain a smaller spot size for different drilling applications [31].

## Figures and Tables

**Table 1:** Specifications of the Nd: YAG laser.

Item	Specification
Maker/Model no.	Qunatel/Brilliant
Maximum energy per pulse	360 mJ/pulse
Repetition rate	10 Hz
(External Repetition rate) [36]	(2–6 M Hz)
Beam diameter of the Nd: YAG rod	6 mm
Pulse duration	6 ns
Power drift	±3%
Max. Power *	1.080 W
Min. Power *	1.020 W
Average power *	1.053 W
Std. Dev. of Power *	7.646 mW
Long-term power stability *	5.60%

Note *: A power meter (Ophir Nova II and Thermopile Sensor: L50(150) A) [32] is used to measure power stability ((Max. Power- Min. Power)/Max. Power) for one hour.

**Table 2:** Control factors and their levels.

Control Factors		Unit	Level		
			1	2	3
A	Laser energy	(mJ/pulse)	75	110	145
B	Focusing position offset	(mm)	+1	0	−1
C	Drill times	(min)	3	5	7
D	Repetition rate (frequency)	(kHz)	1.8	3	4.2

**Table 3:** Experimental design using L_9_ (3^4^) and four control factors.

Run No.	A	B	C	D	Laser Energy (mJ/Pulse)	Focusing Position Offset (mm)	Drill Time (min)	Repetition Rate (kHz)
1	1	1	1	1	75	+1	3	1.8
2	1	2	2	2	75	0	5	3.0
3	1	3	3	3	75	−1	7	4.2
4	2	1	2	3	110	+1	5	4.2
5	2	2	3	1	110	0	7	1.8
6	2	3	1	2	110	−1	3	3.0
7	3	1	3	2	145	+1	7	3.0
8	3	2	1	3	145	0	3	4.2
9	3	3	2	1	145	−1	5	1.8

**Table 4:** Calculated results of the major multiple qualities.

Quality	Roundness at Total	Hillock Ratio	Taper	Average HAZ
Reference	0.929	0.680	0.1348	7
Run				
1	0.787	0.393	0.0580	0.000
2	0.684	0.556	0.0608	0.250
3	0.881	0.282	−0.0049	0.000
4	0.908	0.334	0.1344	2.750
5	0.716	0.574	0.0881	1.750
6	0.929	0.285	0.0100	0.000
7	0.776	0.680	0.0715	7.000
8	0.837	0.551	0.1348	5.250
9	0.921	0.253	0.0211	2.000

**Table 5:** Grey relational generation of multiple qualities.

Quality	Roundness $X_{1}^{*}$ (k)	Hillock Ratio $X_{2}^{*}$ (k)	Taper $X_{3}^{*}$ (k)	Average HAZ $X_{4}^{*}$ (k)
Reference Δ_oi_	1	1	1	1
Run	LTB	STB	NTB	STB
1	0.42	0.673	0.6564	1
2	0	0.291	0.6369	0.964
3	0.804	0.933	0.8929	1
4	0.915	0.81	0.1095	0.607
5	0.129	0.248	0.4412	0.75
6	1	0.926	1	1
7	0.377	0	0.5603	0
8	0.625	0.302	0.1071	0.25
9	0.967	1	0.9206	0.714

**Table 6:** The deviation sequences of multiple qualities.

	Deviation Sequences			
Run	Δ_1_ (k)	Δ_2_ (k)	Δ_3_ (k)	Δ_4_ (k)
1	0.580	0.327	0.344	0.000
2	1.000	0.709	0.363	0.036
3	0.196	0.067	0.107	0.000
4	0.085	0.190	0.891	0.393
5	0.871	0.752	0.559	0.250
6	0.000	0.074	0.000	0.000
7	0.623	1.000	0.440	1.000
8	0.375	0.698	0.893	0.750
9	0.033	0.000	0.079	0.286
max	1.000	1.000	0.839	1.000
min	0.000	0.000	0.000	0.000

**Table 7:** Calculated results of grey relational analysis with equal weighting factors.

	Roundness	Hillock	Taper	HAZ	Overall Grey Relational Grade * (ζ=0.5)/γ(k)	Order/Rank
Run	$\gamma_{1}$ *(k)*	$\gamma_{2}$ *(k)*	$\gamma_{3}$ *(k)*	$\gamma_{4}$ *(k)*		
1	0.463	0.605	0.565	1.000	0.658	4
2	0.333	0.414	0.552	0.933	0.558	6
3	0.718	0.882	0.807	1.000	0.852	3
4	0.855	0.725	0.334	0.560	0.618	5
5	0.365	0.399	0.444	0.667	0.469	7
6	1.000	0.871	1.000	1.000	0.968	1
7	0.445	0.333	0.504	0.333	0.404	9
8	0.571	0.417	0.333	0.400	0.431	8
9	0.938	1.000	0.849	0.636	0.856	2
*: Weighting factor	0.25	0.25	0.25	0.25		

**Table 8:** Response table for the GRG with equal weighting factors.

Controls Factors	Average Grey Relational Grade by Factor Level			
	Level 1	Level 2	Level 3	Max.-Min.
A: Laser energy (mJ/pulse)	0.689 *	0.684	0.563	0.125
B: Focusing position offset (mm)	0.560	0.485	0.891 *	**0.406 #**
C: Drill time (min)	0.685 *	0.677	0.574	0.110
D: Repetition rate (Freq. kHz)	0.660 *	0.643	0.633	0.027

*: maximal level effect for each control factor. #: The control factor with most contribution of level effect.

**Table 9:** Comparison of confirmation experimental results and optimal multiple qualities (Run 6).

Ex NO.	Roundness at Total	Hillock Ratio	Taper	Average HAZ
Remark	The larger the better	The smaller the better	0 degree is better	The smaller the better
Run 6 (A2B3C1D2)	0.929	0.285	0.0100	0.00
Confirmation experiment (A1B3C1D1)	0.938	0.246	−0.0036	0.00

## Data Availability

No applicable.

## Appendix A

This appendix provides the original measured data from the experimental results and the calculation results of the major qualities according to Equations (1)–(8).

**Table A1:** Measured data and quality calculation results for roundness with elliptical approximation.

	Longer Diameter at Entrance Side a_e_ (mm)	Shorter Diameter at Entrance Side b_e_ (mm)	b_e_/a_e_ at Entrance Side *E_en_*	Longer Diameter at Exit Side a_x_ (mm)	Shorter Diameter at Exit Side b_x_ (mm)	b_x_/a_x_ at Exit Side *E_ex_*	Average Roundness
Run							
1	0.927	0.655	0.706	0.566	0.491	0.868	0.787
2	0.630	0.470	0.746	0.298	0.185	0.622	0.684
3	0.756	0.615	0.813	0.699	0.663	0.948	0.881
4	1.294	1.170	0.904	0.342	0.312	0.912	0.908
5	1.067	0.825	0.773	0.784	0.516	0.658	0.716
6	0.763	0.690	0.904	0.519	0.495	0.954	0.929
7	0.750	0.597	0.797	0.315	0.238	0.756	0.776
8	1.119	1.045	0.934	0.424	0.314	0.741	0.837
9	1.179	1.019	0.864	0.801	0.783	0.978	0.921

**Table A2:** Measured data and quality calculation results for average Hillock ratio.

Run	Outer Diameter at Entrance Side O_e_	Inner Diameter at Entrance Side *I_e_*	Hillock Ratio at Entrance Side *Hill_en_*	Outer Diameter at Exit Side *O_x_*	Inner Diameter Average Exit Side *Ix*	Hillock Ratio at Exit Side *Hill_ex_*	Average Hillock Ratio *Hill_avg_*
1	1.069	0.803	0.249	0.980	0.454	0.536	0.393
2	1.167	0.651	0.442	0.866	0.286	0.669	0.556
3	1.253	0.825	0.341	1.099	0.855	0.222	0.282
4	1.660	1.285	0.226	0.858	0.478	0.442	0.334
5	1.869	1.049	0.439	1.791	0.520	0.710	0.574
6	1.039	0.741	0.287	0.950	0.681	0.284	0.285
7	1.818	0.752	0.586	1.430	0.324	0.774	0.680
8	1.682	1.109	0.341	1.260	0.300	0.762	0.551
9	1.601	1.186	0.259	1.408	1.060	0.247	0.253

**Table A3:** Measured data and quality calculation results for taper.

Run	Average Inner Diameter at Entrance Side (*I_e_*)	Average Inner Diameter at Exit Side (*I_e_*)	Taper T_tap_ (*I_e_* − *I*_x_)/2S
1	0.803	0.454	0.522
2	0.651	0.286	0.547
3	0.825	0.855	−0.044
4	1.285	0.478	1.210
5	1.049	0.520	0.793
6	0.741	0.681	0.090
7	0.752	0.324	0.643
8	1.109	0.300	1.213
9	1.186	1.060	0.190

**Table A4:** Measured data and quality calculation results for HAZ.

Run	HAZ at Entrance Side *Haz_en_* (mm)	HAZ at Exit Side *Haz_ex_* (mm)	Average HAZ_avg_ (mm)
1	0.0	0	0
2	0.0	0	0
3	0.0	0	0
4	3.0	2.5	2.75
5	2	1.5	1.75
6	0	0	0
7	7.5	6.5	7
8	5.5	5	5.25
9	1.5	2.5	2

**Table A5:** Confirmation experimental results for roundness.

	Longer Diameter at Entrance Side a_e_ (mm)	Shorter Diameter at Entrance Side b_e_ (mm)	b_e_/a_e_ at Entrance Side *E_en_*	Longer Diameter at Exit Side a_x_ (mm)	Shorter Diameter at Exit Side b_x_ (mm)	b_x_/a_x_ at Exit Side *E_ex_*	Average Roundness
Confirmation results (A1B3C1D1)	0.709	0.629	0.887	0.585	0.578	0.988	0.938

**Table A6:** Confirmation experimental results for average Hillock ratio.

Ex NO.	Outer Diameter at Entrance Side *O_e_*	Inner Diameter at Entrance Side *I_e_*	Hillock Ratio at Entrance Side *Hill_en_*	Outer Diameter at Exit Side *O_x_*	Inner Diameter Average Exit Side *I_e_*	Hillock Ratio at Exit Side *Hill_ex_*	Average Hillock Ratio *Hill_avg_*
Confirmation results (A1B3C1D1)	1.086	0.699	0.356	0.834	0.721	0.136	0.246

**Table A7:** Confirmation experimental results for taper.

Ex NO.	Average Inner Diameter at Entrance Side *(Ie*)	Average Inner Diameter at Exit Side *(I_x_)*	Taper *T_tap_* (*I_e_ − I_x_*)/2S
Confirmation experiment (A1B3C1D1)	0.699	0.721	−0.0036

**Table A8:** Confirmation experimental results for *HAZ_avg_*.

Ex NO.	HAZ at Entrance Side *Haz_en_* (mm)	HAZ at Exit Side *Haz_ex_* (mm)	Average *HAZ_avg_* (mm)
Confirmation experiment (A1B3C1D1)	0	0	0
